# Optimal strategy for linkage of datasets containing a statistical linkage key and datasets with full personal identifiers

**DOI:** 10.1186/1472-6947-14-85

**Published:** 2014-09-25

**Authors:** Lee K Taylor, Katie Irvine, Renee Iannotti, Taylor Harchak, Kim Lim

**Affiliations:** 1 Centre for Epidemiology and Evidence, NSW Ministry of Health, Sydney, Australia; 2 Centre for Health Record Linkage, NSW Ministry of Health, Sydney, Australia

**Keywords:** Data linkage, Record linkage, SLK-581, Linkage methods

## Abstract

**Background:**

Linkage of aged care and hospitalisation data provides valuable information on patterns of health service utilisation among aged care service recipients. Many aged care datasets in Australia contain a Statistical Linkage Key (SLK-581) instead of full personal identifiers. We linked hospital and death records using a full probabilistic strategy, the SLK-581, and three combined strategies; and compared results for each strategy.

**Methods:**

Linkage of Admitted Patient Data for 2000–01 to 2008–09 and Registry of Births, Deaths and Marriages death registration data for 2008–09 for New South Wales, Australia, was carried out using probabilistic methods and compared to links created using four strategies incorporating a SLK-581. The Basic SLK-581 strategy used the SLK-581 alone. The Most Recent SLK-581, Most Frequent SLK-581, and Any Match SLK-581 strategies leveraged probabilistic links between hospital records drawn from the Centre for Health Record Linkage Master Linkage Key. Rates of hospitalisations among people who died were calculated for each strategy and a range of health conditions.

**Results:**

Compared to full probabilistic linkage, the basic SLK-581 strategy produced substantial rates of missed links that increased over the study period and produced underestimates of hospitalisation rates that varied by health condition. The Most Recent SLK-581, Most Frequent SLK-581, and Any Match SLK-581 strategies resulted in substantially lower rates of underestimation than the Basic SLK-581. The Any Match SLK-581 strategy gave results closest to full probabilistic linkage.

**Conclusions:**

Hospitalisation rates prior to death are substantially underestimated by linkage using a SLK-581 alone. Linkage rates can be increased by combining deterministic methods with probabilistically created links across hospital records.

## Background

Linkage of data collected from hospital and aged care services has been used to assess aged care service utilisation [1] and mortality [2] of clients of aged care services in Australia. Information derived from these linked data is essential for policy development and planning to deliver high quality care for older Australians. Creating reliable and accurate linkages poses a challenge as limited personal identifiers are available on aged care services databases in Australia.

The Home and Community Care (HACC) Program, an Australian Government initiative, provides essential community care and support services to enable people who are frail or disabled and at risk of long-term residential care to remain in the community. During 2009–10 there were over 244 302 HACC clients in New South Wales (NSW), with 80% being over 65 years of age [3]. The HACC Minimum Data Set (MDS) is a person based dataset containing information about clients receiving services by HACC providers and the level and type of services the clients receive. The HACC MDS contains a nationally agreed set of data items collected by all HACC service providers for each of their clients [4].

Personally identifying information such as name, address, date of birth and gender are required to perform high quality probabilistic record linkage. However, the identifiable information contained in the HACC MDS is in the form of a statistical linkage key (SLK), the SLK-581. The SLK-581 is a concatenation of 14 characters consisting of the 2^nd^, 3^rd^ and 5^th^ letters of family names, the 2^nd^ and 3^rd^ letters of given names, date of birth in the form DDMMYYYY and a single character for sex. Dummy characters are used for insufficient and missing data so the SLK-581 always has a length of 14 characters [5]. It is not known the extent to which linkage of HACC data to other databases will be adversely affected by the use of the SLK-581 rather than full personal identifiers. Quantification of this effect is important for accurately interpreting the results of future research based on linked records concerning HACC clients.

The Australian Institute of Health and Welfare (AIHW) has shown that the likelihood of common SLK-581 linkage keys for different individuals in large aged care data sets is very low. In a national database of nursing home residents 0.6% of 440 000 people had a non-unique SLK-581 [6]. It was also shown that incomplete data affects between 3% and 4% of SLK-581 linkage keys in the quarterly HACC MDS for the September and December quarters 2002 [4]. This indicates that linkage using the SLK-581 would be expected to have a low rate of incorrect links for a person, that is, a high specificity. The AIHW also linked data of the Aged and Community Care Management Information Systems to the National Death Index and showed that an SLK-581 based linkage strategy had a positive predictive value of 99.8% and a sensitivity of 88.4% compared to a name-based linkage [1]. Bass et al. investigated two SLKs in Western Australia and obtained different results depending on the linkage key and the outcome of interest; the study also found diminishing accuracy over time, the presence of multiple keys for individuals and multiple individuals sharing the same key [7]. Various methods have been developed to improve the sensitivity of the SLK-581 by including event-based and geographic information in the linkage strategy [8-10].

New South Wales (NSW) has the largest population of all Australian States and Territories, comprising 7.2 million people (32% of the Australian population) [11]. We linked NSW Admitted Patient Data (APD) and Registry of Births, Deaths and Marriages (RBDM) death registration data using a full probabilistic strategy, the SLK-581, and three combined strategies. We used the RBDM death data to serve as a proxy for the HACC MDS as both datasets are person based and share a similar age structure. The aims of this study were to: examine the quality of SLK-581 derived for APD data; determine the optimal strategy to be used when linking datasets containing an SLK-581 with datasets containing full personal identifiers; and quantify the extent to which the recommended optimal strategy biases estimates of hospitalisation rates among HACC clients for a range of health priority areas.

## Methods

Ethical approval was obtained from the NSW Population and Health Services Research Ethics Committee.

### Data sources

The APD represents a census of all admitted patient services in NSW. It covers demographic and episode related data for every inpatient that is separated (discharged, transferred or died) from any public, private, repatriation hospital, private day procedure centre or public nursing home in NSW. The APD contains demographic data items, administrative items and coded information. Principal diagnosis and co-morbidities on the APD are coded according to the ICD-10-AM [12]. APD records for the period 1 July 2000 to 30 June 2009 (*n* = 19 874 083) were used for this study.

Deaths occurring in NSW must be notified to the NSW Registry of Births, Deaths and Marriages (RBDM) under the Births, Deaths and Marriages Registration Act 1995. NSW death registration data comprise records of persons who died in NSW, regardless of their place of residence. RBDM death records for the period 1 July 2008 to 30 June 2009 (*n* = 47 110) were used for this study.

### Record linkage and dataset preparation

An SLK-581 was assigned to each record in the APD and RBDM datasets. An SLK-581 was defined as "incomplete" where any component was missing in the source data. January 1 birthdays and age greater than 110 years were considered to be invalid dates of birth. Sex was regarded as invalid if it was missing or took a value other than male or female [5]. Linked datasets were created using five linkage strategies:

#### Strategy 1 - full probabilistic model

Linked records were drawn from the Centre for Health Record Linkage (CHeReL) [13] Master Linkage Key (MLK) [14], which comprises over 45 million records containing personal and demographic information, but no health information, from a range of population-based health and health-related data collections. The MLK is created using a best practice approach in privacy preserving record linkage [15] and the open source probabilistic record linkage software ChoiceMaker [16]. The CHeReL uses the following information to link records for the same person: full name, address, sex, date of birth, country of birth, hospital code, medical record number, hospital dates of admission and discharge, hospital transferred to, hospital transferred from, and date of death. ChoiceMaker uses ‘blocking’ and ‘scoring’ to identify definite and possible matches. During blocking ChoiceMaker searches the target datasets for records that are possible matches to each other. There are two types of blocking: exact blocking requires records to have the same set of valid fields and the same values for these fields; automated blocking builds a set of conditions to find as many records as possible that potentially match each other. Scoring employs a combination of a probabilistic decision, computed using a machine learning technique, and absolute rules, including upper and lower probability cut-offs, to determine the final decision as to whether each match denotes or possibly denotes the same person.

For the entire linked dataset the CHeReL reported the linkage quality as less than 5/1000 missed links and 3/1000 false positive links. Where duplicate RBDM records were found, one record was randomly selected and retained for all five linkage strategies, giving 46 949 records for analysis. The full probabilistic linkage strategy resulted in 44 804 RBDM records, each representing one person, linked to 598 828 APD records; 2145 (4.6%) RBDM records did not link to an APD record.

#### Strategy 2 - basic SLK-581

Linkage of APD to RBDM records was carried out using the SLK-581 only. This strategy resulted in 41 633 RBDM records, each representing one person, linked to 432 026 APD records; 5316 (11.3%) RBDM records did not link to an APD record.

#### Strategy 3 - most recent SLK-581

Each individual identified in the full probabilistic model (Strategy 1) was assigned the SLK-581 from their most recent hospital separation. In the case of incomplete personal identifiers, the most recent complete SLK-581 was used. Where the individual had no records with a complete SLK-581 the most recent incomplete SLK-581 was used. This strategy resulted in 41 081 RBDM records, each representing one person, linked to 555 243 APD records; 5868 (12.5%) RBDM records did not link to an APD record.

#### Strategy 4 - most frequent SLK-581

Each individual identified in the full probabilistic model (Strategy 1) was assigned the complete SLK-581 that occurred most frequently in their hospital separations. Where multiple versions of the SLK-581 occurred with equal greatest frequency, the SLK-581 from the most recent hospital separation was used. Where the individual had no complete SLK-581 the most frequently occurring incomplete SLK-581 was used. This strategy resulted in 41 044 RBDM records, each representing one person, linked to 556 723 APD records; 5905 (12.6%) RBDM records did not link to an APD record.

#### Strategy 5 - any match SLK-581

This involved a two stage linkage process. First, each RBDM death record was linked with the APD using the SLK-581. Second, APD records that linked to an RBDM record were drawn from the CHeReL Master Linkage Key using the method described for the full probabilistic model. This strategy resulted in 41 633 RBDM records, each representing one person, linked to 571 923 APD records; 5316 (11.3%) RBDM records did not link to an APD record.

### Analysis

A descriptive analysis of the quality of the SLK-581 in the APD and RBDM death registration data was carried out. The proportion of records with missing data and possibly poor quality data [5] for the various components of the SLK-581 was assessed; for the APD this was also assessed by whether the records related to public or private hospitals.

The proportion of persons with at least one missed link or at least one false positive link were examined for strategies 2 to 5 using strategy 1 (Full Probabilistic Model) as the standard. As one individual could have more than one linked hospital record using Strategy 1, it was possible for an individual to simultaneously have both false positive and missed links according to strategies 2 to 5. The trend in the proportion of persons who died with either at least one missed link or at least one false positive link to the APD was examined by year of death.

We examined the proportion of persons who died in NSW between 1 July 2008 and 30 June 2009 inclusive that were hospitalised prior to death by linkage strategy and year of death. Mean and median number of hospitalisations and 95% confidence intervals were calculated.

For each linkage strategy, the number of hospitalisations among persons who died in NSW during 2008–09 was examined for a range of health priority areas (cancer, cardiovascular disease, mental health, asthma, diabetes, arthritis and musculoskeletal conditions, and injury prevention) and ambulatory care sensitive conditions [17]. Health priority areas were selected using the relevant International Classification of Disease (ICD-10-AM) codes [see Additional file 1]. Hospitalisations prior to death were analysed over nine time periods from the one year period from 1 July 2008 to 30 June 2009 to the nine year period from 1 July 2000 to June 2009. Analyses were carried out using SAS 9.2 [18].

## Results

**Figure 1:**
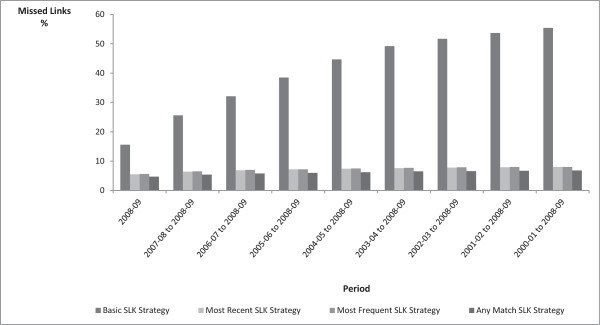
**Persons with at least one missed link to an APD record compared to the full probabilistic linkage strategy, by type of SLK-581 strategy.** SLK: Statistical Linkage Key.

**Figure 2:**
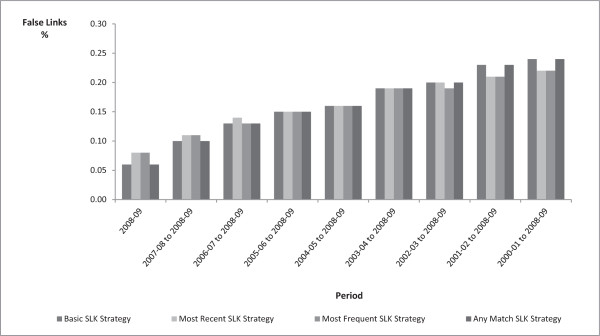
**Persons with at least one incorrect link to an APD record compared to the MLK Strategy, by type of SLK-581 strategy.** MLK: Master Linkage Key. SLK: Statistical Linkage Key.

Of the 19.9 million APD records, 12.9 million (65.1%) related to public hospital admissions and 6.9 million (34.9%) to private hospital admissions (Table 1). Over the nine-year study period 32% of APD records had an incomplete SLK-581—11% of public hospital records and 71% of private hospital records. Over 99% of records with incomplete SLK-581 had incomplete name information. The proportion of private hospital APD records with missing name information decreased from 80% in 2000–01 to 62% in 2008–09; no trend in missing name was observed for public hospital records. Of the 47 110 RBDM death records sourced for the project, 265 (0.6%) had an incomplete SLK-581.Compared to the Full Probabilistic Model, the percentage of persons with at least one missed link varied between the SLK-581 strategies. The Basic SLK-581 Strategy produced the highest percentage of persons with missed links, increasing from 15.6% to 55.4% over the 9-year study period; while the Any Match SLK-581 Strategy produced the lowest rate of persons with missed links, increasing from 4.7% to 6.8% over the same period (Figure 1). The rates of persons with missed links for the Most Recent SLK-581 and Most Frequent SLK-581 increased from 5.5% to 8.0% and 5.6% to 8.0% respectively over the study period. All four Strategies resulted in very small percentages of persons with false positive links at less than 0.25% for all Strategies over the study period, with almost no variation between the Strategies (Figure 2).

**Table 1:** Components contributing to incomplete SLK-581 by data source

**Component**	**Admitted patient records**						**Death records**	
	**Public hospitals**		**Private hospitals**		**All hospitals**			
	**(*n*** **= 12 932 344)**		**(*n*** **= 6 941 739)**		**(*n*** **= 19 874 083)**		**(*n*** **= 47 110)**	
	**No.**	**%**	**No.**	**%**	**No.**	**%**	**No.**	**%**
Age over 110 years	77	0.0	2	0.0	79	0.0	1	0.0
Missing or invalid date of birth	69 606	0.5	25 079	0.4	94 685	0.5	257	0.6
Missing given name and surname	1 404 099	10.9	4 935 799	71.1	6 339 898	31.9	0	0.0
Missing surname only	5	0.0	1413	0.0	1418	0.0	4	0.0
Missing given name only	127	0.0	12 485	0.2	12 612	0.1	0	0.0
Missing or invalid sex	1015	0.0	26	0.0	1041	0.0	4	0.0
Total incomplete SLK-581	1 464 764	11.3	4 957 467	71.4	6 422 231	32.3	265	0.6

SLK: Statistical Linkage Key.

Compared to the Full Probabilistic Model all four SLK-581 Strategies underestimated the proportion of individuals hospitalised at least once prior to death by 6%-7% (Table 2). The proportions were similar between the four SLK-581 strategies and the level of under-estimation was consistent regardless of the number of years of linked APD data used in the analysis.

**Table 2:** Persons who died in 2008–09: percentage hospitalised by years prior to death and linkage strategy

**Years prior to death**	**Full probabilistic model**	**SLK-581**			
		**Basic**	**Most recent**	**Most frequent**	**Any match**
	**%**	**%**	**%**	**%**	**%**
2008-09	75.1	67.8	69.6	69.5	67.8
2007-08 to 2008-09	84.3	76.9	77.9	77.8	76.9
2006-07 to 2008-09	88.4	81.0	81.5	81.4	81.0
2005-06 to 2008-09	90.8	83.5	83.7	83.6	83.5
2004-05 to 2008-09	92.4	85.1	85.0	84.9	85.1
2003-04 to 2008-09	93.5	86.3	85.9	85.8	86.3
2002-03 to 2008-09	94.4	87.3	86.6	86.5	87.3
2001-02 to 2008-09	95.0	88.1	87.2	87.1	88.1
2000-01 to 2008-09	95.4	88.7	87.5	87.4	88.7

SLK: Statistical Linkage Key.

The four SLK-581 Strategies underestimated the mean (Table 3) and median (Table 4) number of hospitalisations prior to death compared to the Full Probabilistic Model. For the mean number of hospitalisations prior to death, the underestimation of hospitalisations relative to the Full Probabilistic Model increased with increasing study period for the Basic, the Most Recent and the Most Frequent SLK-581 Strategies; while the relative underestimation decreased with increasing study period for the Any Match SLK-581 Strategy. The Basic SLK-581 Strategy underestimated mean hospitalisations by 16% for a one year study period increasing to 28% over nine years; the Most Recent and the Most Frequent SLK-581 Strategies underestimated hospitalisations by 4% for one year increasing to 8% for nine years; and the Any Match SLK-581 Strategy underestimated hospitalisations by 8% for one year decreasing to 5% for nine years relative to the Full Probabilistic Model. For the median number of hospitalisations prior to death, there was little variation in results across the linkage Strategies over shorter periods of time; however, over nine years the Any Match SLK-581 Strategy gave results closest to the Full Probabilistic Model and the Basic SLK-581 Strategy gave the greatest underestimation of the Full Probabilistic Model results.

**Table 3:** Persons who died in 2008–09: mean hospitalisations by years prior to death and linkage strategy

**Years**	**Full probabilistic model**		**SLK-581 strategy**							
			**Basic**		**Most recent**		**Most frequent**		**Any match**	
	**Mean**	**(95% CI)**	**Mean**	**(95% CI)**	**Mean**	**(95% CI)**	**Mean**	**(95% CI)**	**Mean**	**(95% CI)**
2008-09	2.5	(2.5-2.6)	2.1	(2.0-2.1)	2.4	(2.3-2.4)	2.4	(2.3-2.4)	2.3	(2.2-2.4)
2007-08 to 2008-09	5.0	(4.8- 5.1)	3.9	(3.8- 4.0)	4.6	(4.5- 4.8)	4.6	(4.5- 4.8)	4.6	(4.5-4.7)
2006/07 to 2008/09	6.8	(6.5- 7.0)	5.3	(5.1- 5.5)	6.3	(6.1- 6.5)	6.3	(6.1- 6.5)	6.3	(6.1-6.5)
2005/06 to 2008/09	8.2	(7.9- 8.5)	6.2	(6.0- 6.5)	7.6	(7.3- 7.9)	7.6	(7.3-7.9)	7.7	(7.4-7.9)
2004/05 to 2008/09	9.4	(9.0- 9.7)	6.9	(6.6- 7.2)	8.7	(8.4- 9.0)	8.7	(8.4- 9.1)	8.8	(8.5-9.1)
2003/04 to 2008/09	10.4	(10.1-10.8)	7.5	(7.2 -7.8)	9.7	(9.3-10.1)	9.7	(9.4- 10.1)	9.8	(9.5-10.2)
2002/03 to 2008/09	11.3	(10.9-11.8)	8.2	(7.9- 8.5)	10.5	(10.1-10.9)	10.5	(10.2-11.0)	10.7	(10.3-11.1)
2001/02 to 2008/09	12.1	(11.7-12.5)	8.7	(8.4- 9.1)	11.2	(10.8-11.7)	11.3	(10.8-11.7)	11.4	(11.0-11.9)
2000/01 to 2008/09	12.8	(12.3-13.2)	9.2	(8.8-9.6)	11.8	(11.4-12.3)	11.9	(11.4-12.3)	12.1	(11.6-12.5)

SLK: Statistical Linkage Key.

**Table 4:** Persons who died in 2008–09: median hospitalisations by years prior to death and linkage strategy

**Years**	**Full probabilistic model**		**SLK-581 strategy**							
			**Basic**		**Most recent**		**Most frequent**		**Any match**	
	**Median**	**(25,75 quartiles)**	**Median**	**(25,75 quartiles)**	**Median**	**(25,75 quartiles)**	**Median**	**(25,75 quartiles)**	**Median**	**(25,75 quartiles)**
2008/09	1	(1–3)	1	(0–2)	1	(0–3)	1	(0–3)	1	(0–3)
2007/08 to 2008/09	3	(1–5)	2	(1–4)	2	(1–5)	2	(1–5)	2	(1–5)
2006/07 to 2008/09	3	(1–7)	3	(1–5)	3	(1–6)	3	(1–6)	3	(1–6)
2005/06 to 2008/09	4	(2–8)	3	(1–6)	4	(1–7)	4	(1–7)	4	(1–8)
2004/05 to 2008/09	5	(2–9)	3	(1–7)	4	(2–8)	4	(2–8)	5	(2–9)
2003/04 to 2008/09	6	(3–10)	4	(2–7)	5	(2–9)	5	(2–9)	5	(2–9)
2002/03 to 2008/09	6	(3–11)	4	(2–8)	6	(2–10)	6	(2–10)	6	(2–10)
2001/02 to 2008/09	7	(3–11)	4	(2–8)	6	(3–11)	6	(3–11)	6	(3–11)
2000/01 to 2008/09	7	(4–12)	5	(2–9)	6	(3–12)	6	(3–12)	7	(3–12)

SLK: Statistical Linkage Key.

Compared to the Full Probabilistic Model, the basic SLK-581 Strategy substantially underestimated the proportion of persons hospitalised prior to death across all eight of the health conditions examined (Table 5). The Any Match SLK-581 Strategy gave results closest to the Full Probabilistic Model, underestimating persons hospitalised by 6% or less for all conditions. The Most Recent and the Most Frequent SLK-581 Strategies provided almost identical results, with underestimation ranging from 6% (for asthma and cancer) to 8% (for mental illness). Underestimation of persons hospitalised prior to death with the Basic SLK-581 Strategy ranged from 9% for diabetes to 31% for arthritis.

**Table 5:** Persons who died in 2008–09: hospitalisations prior to death by condition and linkage strategy

**Condition**	**Full probabilistic model**	**SLK-581 strategy**			
		**Basic**	**Most recent**	**Most frequent**	**Any match**
Rate (%)					
Cancer	36.1	28.3	33.8	33.8	34.2
Cardiovascular disease	47.4	41.5	44.2	44.1	44.8
Mental Illness	10.1	8.9	9.3	9.3	9.6
Asthma	1.2	1.0	1.1	1.1	1.1
Arthritis	19.7	13.7	18.4	18.3	18.6
Diabetes	21.2	19.1	19.7	19.6	20.0
Injury	42.2	37.7	39.1	39.1	39.8
Ambulatory care sensitive conditions	47.3	41.3	44.2	44.1	44.8
Rate ratio					
Cancer	1.00	0.78	0.94	0.94	0.95
Cardiovascular disease	1.00	0.88	0.93	0.93	0.95
Mental Illness	1.00	0.88	0.92	0.92	0.95
Asthma	1.00	0.86	0.94	0.94	0.96
Arthritis	1.00	0.69	0.93	0.93	0.95
Diabetes	1.00	0.91	0.93	0.93	0.94
Injury	1.00	0.89	0.93	0.93	0.95
Ambulatory care sensitive conditions	1.00	0.87	0.93	0.93	0.95

SLK: Statistical Linkage Key.

## Discussion

We compared four strategies for linkage of NSW APD data to RBDM death data using the SLK-581 with linkage using a Full Probabilistic Model. The Basic SLK-581 Strategy produced substantial rates of missed links that increased from 16% to 56% over the 9-year study period. The remaining three SLK-581 strategies leveraged the probabilistic links in the MLK to enhance the deterministic SLK-581 linkages, substantially reducing the rate of missed links, with the Any Match SLK-581 Strategy producing the lowest rate of missed links. When compared with probabilistic linkage, all SLK-581 Strategies produced less than 0.25% false positive links. The basic SLK-581 Strategy substantially under-estimated the proportion of persons hospitalised prior to death for a range of priority conditions; the Any Match SLK-581 Strategy produced results that were closest to the Full Probabilistic Model.

The strength of this study is that we used a large linked population-based dataset for NSW, covering about one third of the Australian population. The CHeReL MLK is created using probabilistic record linkage techniques. The quality of linkage is checked continuously and is maintained so that there are no more than 5 per 1000 false positive links and no more than 5 per 1000 missed links [19], making it an appropriate standard against which to assess the performance of the SLK-581. The SLK-581 was incomplete for 32% of APD records and less than 1% of death records, due largely to missing information on name. We found that the rate of missed links was higher in patients admitted for treatment of arthritis than for other conditions. While not investigated as part of this study, in Australia these patients are more likely to be admitted for elective joint replacement to private hospitals that have higher rates of missing name information than public hospitals. The increase in linkage rates that we observed by leveraging the probabilistic links in the MLK would be less for linkage of datasets with higher proportions of records with a complete SLK-581. The results are therefore generalisable to datasets with a substantial proportion of records with incomplete information on names, dates of birth and/or sex.

The SLK-581 has previously been shown to under-estimate mortality and length of hospital stay compared to a full probabilistic linkage [7], and to have low rates of false positive links [6]. The extent to which linkage using the SLK-581 under-estimates the occurrence of health events depends on the quality and completeness of the personal information that contributes to the components of the SLK-581. A stepwise deterministic record linkage algorithm using the SLK-581 and additional information on area of residence and date of death has been shown to identify links with a positive predictive value of 99.7% and a sensitivity of 98.5% when compared with a probabilistic record linkage approach [2]. The stepwise approach relies heavily on the availability of first name and/or surname, both of which were missing in about one third of APD records in our linked dataset, and would have contributed little to this study.

Personal identifiers may be collected in a limited way in order to protect individual privacy. Linkage of large administrative datasets that include both limited and full personal identifiers may re-identify individuals and therefore pose a risk to privacy. The CHeReL holds only personal identifiers for the purpose of linkage and does not hold health information. Once the linkage is completed by the CHeReL, linkage ‘keys’ comprising the dataset record number and a project person number (PPN) obtained from the linkage are returned to each data custodian, who in turn add the PPN to the source dataset and provide the health information and PPN to the investigators for analysis. This process separates the linkage (which requires the use of personal identifiers) from the analysis (which does not) and provides a robust mechanism for preserving individual privacy [15].

It would be possible to increase linkage rates by improving the quality of reporting of personal identifiers, such as name, on the source datasets. It is likely that the addition of further links from other population-based datasets would further improve the performance of a combined strategy.

Our study measured the extent to which deterministic links created using an SLK-581 can be improved by leveraging links created using full probabilistic techniques and linked population based data currently available in NSW. The results of this study indicate that a combined approach of deterministic linkage using an SLK-581 and probabilistic linkage of APD records provides more accurate results than using the SLK-581 alone. While use of the SLK-581 is unique to Australia, other deterministic linkage approaches using limited personal identifiers are possible such as linkage using the first two letters of the first name and surname, sex and date of birth. A combined approach using both probabilistic and deterministic linkage methods could be used in other settings where the datasets to be linked include full personal identifiers on some datasets and limited personal identifiers on others.

## Conclusions

Where datasets contain high proportions of missing name information, deterministic linkage using a SLK-581 results in a substantial number of missed links associated with a low rate of false positive links. By leveraging existing links in a population-based linked dataset that has been created using a full probabilistic approach, the number of missed links can be substantially reduced, and the associated under-reporting of measures of population health and health service utilisation mitigated, while maintaining a low rate of false positive links.

## Competing interests

The authors declare that they have no competing interests.

## Authors’ contributions

KI conceptualised the study. RI, TH and KL carried out the statistical analysis. All authors were involved in study design and manuscript preparation. All authors read and approved the final manuscript.

## Pre-publication history

The pre-publication history for this paper can be accessed here:

http://www.biomedcentral.com/1472-6947/14/85/prepub

## Supplementary Material

Additional file 1 ICD-10-AM codes for selecting health priority areas and ambulatory care sensitive conditions (ACSC) associated with hospitalisation. Click here for file
