# Supplementary material for: Optimal strategy for linkage of datasets containing a statistical linkage key and datasets with full personal identifiers
Source: BMC Med Inform Decis Mak. 2014 Sep 25;14:85. doi: 10.1186/1472-6947-14-85 (PMC4236530; doi:10.1186/1472-6947-14-85)
Supplement: Additional file 1 — ICD-10-AM codes for selecting health priority areas and ambulatory care sensitive conditions (ACSC) associated with hospitalisation. [file 1472-6947-14-85-S1.docx]

Additional file

ICD-10-AM codes for selecting health priority areas and ambulatory care sensitive conditions (ACSC) associated with hospitalisation.

ICD-10-AM codes for selecting health priority areas and ambulatory care sensitive conditions (ACSC) associated with hospitalisation

| Group | ICD-10-AM codes | Selection information |
| --- | --- | --- |
| Health priority area | | |
| Cancer | C00 – C96 | Principal diagnosis only |
| Cardiovascular diseases | I00 – I99 | Principal diagnosis |
| Diabetes | E10 – E14 and O24 | In an diagnosis field |
| Mental health | F00 – F99 | Principal diagnosis |
| Asthma | J45, J46 | Principal diagnosis |
| Arthritis and musculoskeletal conditions | M00 – M99 | Principal diagnosis |
| Injury | V00 – V99; W00 – W99; X00 – X99 ; Y00 – Y39; Y85 – Y87; Y89; | External cause |
| Ambulatory care sensitive conditions | | |
| Vaccine preventable | | |
| Influenza and pneumonia | J10, J11, J13, J14, J15.3, J15.4,  J15.7, J15.9, J16.8, J18.1, J18.8 | In any diagnosis field; exclude people under 2 months; ICD-9-CM: exclude cases with secondary diagnosis of 282.6; ICD-10-AM: exclude cases with secondary diagnosis of D57 |
| Other vaccine preventable | A35, A36, A37, A80, B05, B06,  B16.1, B16.9, B18.0, B18.1, B26, G00.0, M01.4 | In any diagnosis field |
| Chronic | | |
| Diabetes complications | E10.0-E10.8, E11.0-E11.8, E12.0- E12.8, E13.0-E13.8, E14.0-E14.8 | Principal diagnosis only |
| Nutritional deficiencies | E40-E43, E55.0, E64.3 | Principal diagnosis only |
| Iron deficiency anaemia | D50.1-D50.9 | Principal diagnosis only |
| Hypertension | I10, I11.9 | Principal diagnosis only; ICD-9-CM: exclude cases with procedure code of 35, 36, 37.5, 37.6, 37.7, 37.8; ICD-10-AM: exclude cases with procedures in blocks 600-693, 705-707, 717 and procedure codes 38721-00, 38721-01, 90226-00 |
| Congestive heart failure | I11.0, I50, J81 | Principal diagnosis only; ICD-9-CM: exclude cases with procedure code of 35, 36, 37.5, 37.6, 37.7, 37.8; ICD-10-AM: exclude cases with procedures in blocks 600-693, 705-707, 717 and procedure codes 38721-00, 38721-01, 90226-00 |
| Angina | I20, I24.0, I24.8, I24.9 | Principal diagnosis only; ICD-9-CM: exclude cases with procedure codes 01 to 86.99; ICD-10-AM: exclude cases with procedure codes in blocks 1-1779 |
| Chronic obstructive pulmonary disease | J41-J44, J47, (J20) | Principal diagnosis only; ICD-9-CM: 466.0 only with secondary diagnosis of 491, 492, 494, 496; ICD-10-AM: J20 only with secondary diagnosis of J41, J42, J43, J44, J47 |
| Asthma | J45, J46 | Principal diagnosis only |
| Acute | | |
| Dehydration and gastroenteritis | E86, K52.2, K52.8, K52.9 | Principal diagnosis only |
| Convulsions and epilepsy | G40, G41, O15, R56 | Principal diagnosis only |
| Ear, nose and throat infections | H66, H67, J02, J03, J06, J31.2 | Principal diagnosis only |
| Dental conditions | A69.0, K02-K06, K08, K09.8, K09.9, K12, K13 | Principal diagnosis only |
| Perforated/bleeding ulcer | K25.0- K25.2, K25.4-K25.6, K26.0-K26.2, K26.4-K26.6, K27.0-K27.2, K27.4-K27.6, K28.0-K28.2, K28.4-K28.6 | Principal diagnosis only |
| Ruptured appendix | K35.0 | In any diagnosis field |
| Pyelonephritis | N10, N11, N12, N13.6 | Principal diagnosis only |
| Pelvic inflammatory disease | N70.0, N70.1, N70.9, N73, N74.0-N74.1, N74.2-N74.8 | Principal diagnosis only |
| Cellulitis | L03, L04, L08.0, L08.8, L08.9, L88, L98.0, L98.3 | Principal diagnosis only; ICD-9-CM: exclude cases with procedure codes 01 to 86.99 except 86.0 where it is the only listed procedure; ICD-10-AM: exclude cases when any procedure performed from blocks 1-1779 except when the following procedures done as the only ones: blocks: 1604-1606, 1608 and procedures: 90660-00, 30207-00, 30676-00, 30679-00, 34530-01 and 47912-00. |
| Gangrene | R02 | In any diagnosis field |
